# Efficacy and safety of postoperative radiotherapy in locally advanced esophageal squamous cell carcinoma patients with pathologic incomplete response after neoadjuvant immunochemotherapy: a retrospective cohort study

**DOI:** 10.3389/fimmu.2025.1660097

**Published:** 2025-11-21

**Authors:** Yiming Mu, Fangjie Ding, Lili Qiao, Xue Wu, Shunshun Bao, Fengjun Liu, Pingping Hu, Yan Zhang, Ning Liang, Jian Xie, Guodong Deng, Yuying Hao, Dianzheng An, Jingxin Zhang, Jiandong Zhang, Yingying Zhang

**Affiliations:** 1 Department of Oncology, The First Affiliated Hospital of Shandong First Medical University and Shandong Provincial Qianfoshan Hospital, Jinan, Shandong, China; 2 Clinical Medical College of Jining Medical University, Jining, China; 3 Shandong Cancer Hospital and Institute, Shandong First Medical University and Shandong Academy of Medical Sciences, Jinan, Shandong, China; 4 Shandong University Cancer Center, Jinan, Shandong, China; 5 Department of Radiation Oncology, Shandong Cancer Hospital and Institute, Shandong First Medical University and Shandong Academy of Medical Sciences, Jinan, Shandong, China; 6 Cheeloo College of Medicine, Shandong University, Jinan, China

**Keywords:** esophageal squamous cell carcinoma, recurrence, neoadjuvant immunotherapy, postoperative radiotherapy, treatment-related adverse events

## Abstract

**Background:**

Neoadjuvant immunotherapy combined with chemotherapy (NICT) has demonstrated a good pathological complete response (pCR) rate and prognosis in locally advanced esophageal squamous cell carcinoma (LA-ESCC). However, the value and safety of postoperative radiotherapy (PORT) in the group that does not achieve pCR remain unclear.

**Methods:**

This retrospective study included LA-ESCC patients with non-pCR after NICT. Propensity score matching (PSM) was used to balance baseline characteristics between the PORT and non-PORT groups. The outcomes assessed were disease-free survival (DFS), recurrence patterns, and treatment-related toxicity.

**Results:**

In the cohort of 204 enrolled patients, 50 underwent PORT, while the remaining 154 did not, with a median follow-up of 27.0 months. 32 (20.8%) of the non-PORT patients experienced recurrence events, including locoregional recurrence (10/32, 31.3%), distant metastasis (10/32, 31.3%), and mixed patterns (12/32, 37.5%), and 71.9% of cases underwent disease progression within 12 months. With regard to patterns of locoregional recurrence, mediastinal lymph node metastasis represented the most prevalent failure pattern. In terms of distant metastasis, supraclavicular lymph node metastasis was the most commonly observed mode. By PSM analysis, DFS was improved for the patients receiving PORT (HR, 0.26; 95% CI, 0.09-0.77; P = 0.008). Subgroup and analyses revealed a significant increase in both 1- and 2-year DFS rates in patients with ypN+, ypT3-4, yp Stage III-IVA, tumor regression grade (TRG) 2-3, non-downstaging of T stage or middle/lower thoracic esophageal tumors. In patients with non-downstaging of N or TNM status, there was a notable enhancement in the 2-year DFS rate. Treatment-related adverse events (TRAEs) were predominantly grade 1–2 in the PORT group, with radiation esophagitis and myelosuppression being the most frequently observed.

**Conclusion:**

Mediastinal and supraclavicular lymph node metastasis remains the primary cause of treatment failure in LA-ESCC patients with non-pCR after NICT and without PORT. PORT significantly improves DFS in patients with high-risk clinicopathological features or poor response to NICT, and demonstrates a favorable safety profile, indicating an effective adjuvant treatment strategy for improving prognosis.

## 1 Introduction

Esophageal cancer was the sixth most common cause of cancer death worldwide in 2020, with a particularly high prevalence in East and Southeast Asia, where esophageal squamous cell carcinoma (ESCC) is the predominant subtype (1, 2). More than two-thirds of patients with ESCC are diagnosed at an advanced local stage (3). The CROSS and NEOCRTEC5010 trials have demonstrated that neoadjuvant chemoradiotherapy (NCRT) is the prevailing standard of care for resectable locally advanced esophageal squamous cell carcinoma (LA-ESCC). Nevertheless, a considerable number of patients exhibit elevated risk for recurrence and subsequent mortality from ESCC (4, 5). Consequently, there is a compelling need to explore novel therapeutic interventions with the aim of further decreasing recurrence rates and improving long-term survival rates.

The advent of immune checkpoint inhibitors (ICIs) has profoundly reshaped the therapeutic landscape of ESCC. Combined with chemotherapy, ICIs have demonstrated remarkable efficacy and safety in locally advanced disease, as evidenced by trials including KEYNOTE-590 and CheckMate 648/577 (6–11). Neoadjuvant immunotherapy combined with chemotherapy (NICT) has further shown enhanced pathological complete response (pCR) rates compared to chemoradiotherapy, without increased toxicities (12, 13). However, the role of postoperative adjuvant therapies, such as postoperative radiotherapy (PORT), remains controversial in non-pCR patients who face high recurrence risks.

This study investigates the recurrence patterns after NICT and evaluates the impact of PORT on outcomes, including subgroup analyses to identify patients who may benefit from intensified local therapy. Safety profiles of PORT were also assessed to guide risk-adapted strategies.

## 2 Methods

### 2.1 Study design and participants

**Figure 1:**
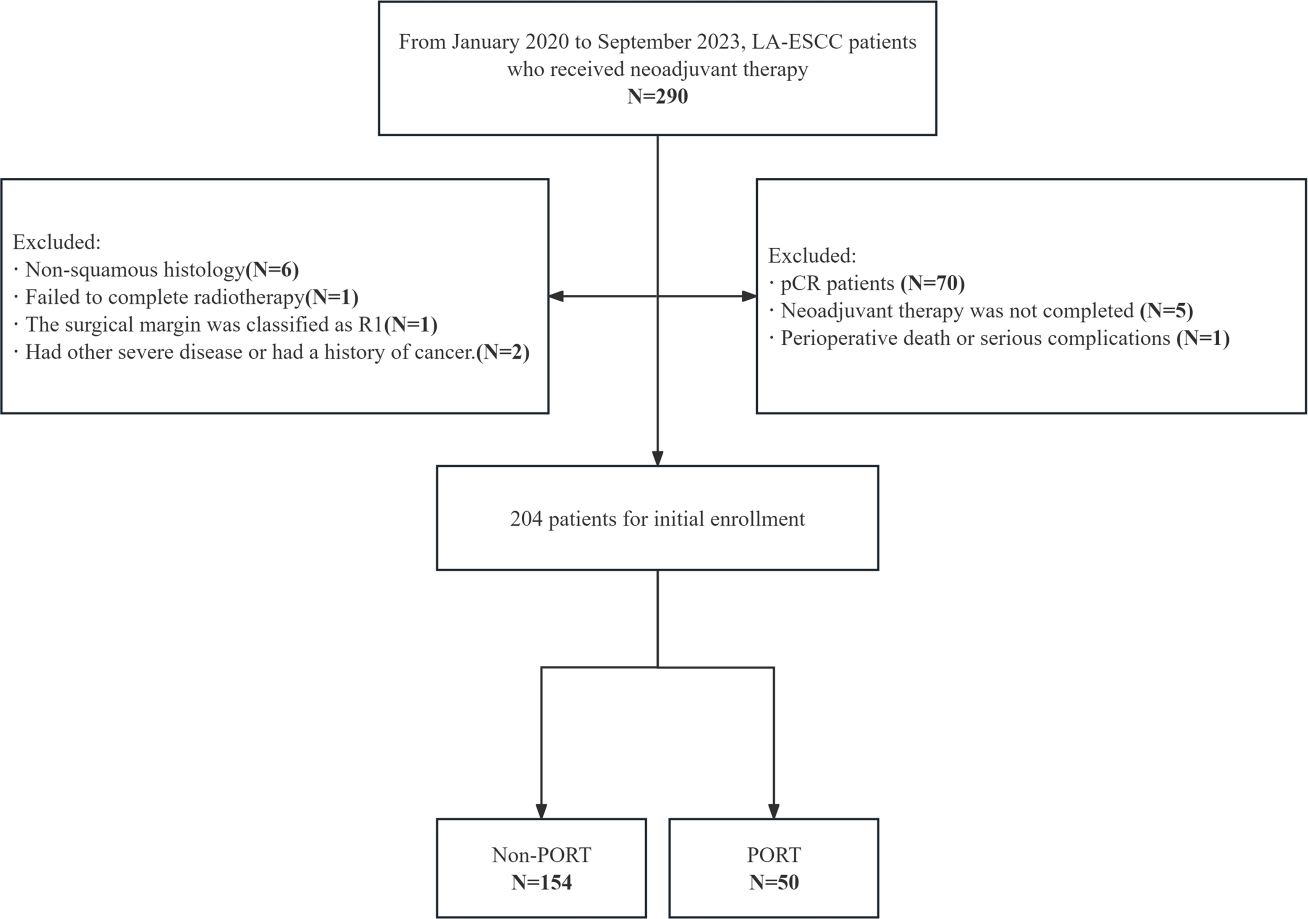
CONSORT flow diagram of patient enrollment. LA-ESCC, local advanced esophageal squamous cell carcinoma; pCR, pathological complete response; PORT, postoperative radiotherapy.

The data of patients with resectable thoracic LA-ESCC who received NICT followed by surgery at the First Affiliated Hospital of Shandong First Medical University and the Shandong Cancer Hospital from January 2020 to September 2023 were analyzed. Inclusion criteria included (a) histopathologically confirmed diagnosis of LA-ESCC; (b) surgery after undergoing NICT; (c) age 18–80 years; (d) Karnofsky performance status ≥70 without severe critical organ dysfunction (heart, lungs, liver, kidneys, and hematologic system). Exclusion criteria were (a) history of prior malignancy; (b) patients with prior neoadjuvant radiotherapy; (c) distant metastases at baseline; (d) patients ineligible for immunotherapy or immunotherapy plus chemotherapy; (e) inability to complete postoperative therapy due to severe complications; (f) active autoimmune disease; (g) poor treatment adherence or incomplete follow-up; and (h) missing clinical data. Data collected included: age, gender, body mass index, KPS score, smoking history, alcohol consumption history, concomitant diseases, family history, neoadjuvant and adjuvant treatment options, surgical approach, American Joint Committee on Cancer (AJCC) staging (8th edition of the staging manual), tumor location, histologic subtype, pathological diagnosis by TNM staging, mode of failure, time to last follow-up, and last status at last follow-up. Written informed consent was not required as the study was retrospective. The study was approved by the Institutional Ethics Review Board (approval number: 2022 S398 and SDTHEC202409044) (**Figure 1**).

### 2.2 Treatment strategy

A comprehensive diagnostic workup was conducted for all patients, including upper abdominal Computed Tomography (CT), Magnetic Resonance Imaging (MRI), Positron Emission Tomography-Computed Tomography (PET-CT), endoscopy, and biopsy for evaluation of primary tumors and lymph nodes. The NICT regimen consisted of 2 to 4 cycles of ICIs (e.g., camrelizumab, sintilimab, pembrolizumab, tislelizumab, or toripalimab, 200 mg every 3 weeks) plus concurrent chemotherapy. The chemotherapy regimen comprised albumin-bound paclitaxel or docetaxel in conjunction with a platinum analog (e.g., nedaplatin, cisplatin, carboplatin, or oxaliplatin), albumin-bound paclitaxel with a fluoropyrimidine analog (e.g., tegafur or 5-fluorouracil), or albumin-bound paclitaxel with a platinum analog and tegafur. Standard radical surgery for LA-ESCC was performed on all patients, with R0 resection defined as the complete removal of the tumor with negative microscopic margins. T, N, and TNM downstaging were defined as the reduction in ypT/ypN/ypTNM stage compared with cT/cN/cTNM stage before NICT. Hospital pathology departments followed the 8th edition of the AJCC guidelines for postoperative staging, categorizing carcinoma *in situ* (CIS) as T1. After surgery, most patients received a combination chemotherapy and immunotherapy regimen, most commonly camrelizumab in combination with albumin-bound paclitaxel or tegafur. Some patients received PORT, and the interval between the surgery and radiotherapy was mainly within 3 months.

### 2.3 PORT technique and target volume delineation

PORT was delivered via intensity-modulated radiotherapy (IMRT). Target volumes were contoured based on postoperative contrast-enhanced CT covering the neck, chest, and abdominal regions, with reference to primary tumor location, surgical approach, preoperative imaging, postoperative pathological stage, and patient status. In general, the clinical target volume (CTV) primarily included the bilateral supraclavicular regions and upper mediastinal regions. For patients with lower thoracic esophageal cancer and ≥3 lymph node metastases, the following lymph node regions may optionally be included based on individual patient conditions: 104, 105, 106, 107 nodal regions, and abdominal 1, 2, 3, and 7 nodal regions (14). The median interval between surgery and PORT initiation was 2.0 months (range: 0.7-5.9 months), with 76.1% completing PORT within 3 months. The total dose of adjuvant radiotherapy was most commonly 45–60 Gy, with a single dose of 1.8 or 2 Gy. All treatment plans maintained dose constraints for organs at risk, including the lungs (Dmean ≤ 15Gy, V5 < 60%, V20 ≤ 30%), heart (Dmean < 26 Gy, V30 < 40%, V40 < 30%), and spinal cord (Dmax ≤ 45 Gy).

### 2.4 Endpoints and follow up

The primary outcomes assessed were disease-free survival (DFS), recurrence patterns, and treatment-related toxicity. DFS was defined as the time from surgery until disease recurrence, progression, death, or the last follow-up. Recurrences were classified as local, distant, or mixed patterns at first recurrence. Locoregional recurrence was defined as recurrence occurring at the anastomosis or regional lymph nodes. Distant metastasis involved distant organs or non-regional lymph nodes (e.g., supraclavicular lymph nodes). Mixed pattern was defined as both locoregional recurrence and distant metastasis. Treatment-related toxicity was assessed using the Common Terminology Criteria for Adverse Events (CTCAE) version 5.0 for grading hematological toxicities, gastrointestinal reactions, and radiotherapy-related toxicities (including radiation esophagitis). Myelosuppression was specifically evaluated according to the World Health Organization (WHO) grading criteria for acute and subacute toxicity of anticancer drugs. Follow-up data were collected through electronic medical records and supplemented by telephone interviews, with the cutoff date for data analysis set as July 30, 2025.

### 2.5 Statistical analysis

Kaplan-Meier (KM) curves were used to estimate disease-free survival (DFS), and all regression analyses reported hazard ratios (HRs) with 95% confidence intervals (CIs). Stepwise Cox regression models (entry criterion: P < 0.05) incorporated variance inflation factors (VIFs) to assess multicollinearity. Subsequently, data underwent 1:2 propensity score matching (PSM; caliper= 0.2×SD) for key clinicopathological variables significantly associated with baseline characteristics and prognosis, and HRs and corresponding p-values from Cox models were calculated based on the matched data. Subgroup analyses were performed using truncated Cox proportional hazards models to calculate differences in 1-year and 2-year DFS. Safety-related categorical variables were analyzed using chi-square or Fisher’s exact tests. All analyses were performed using R 4.4.0.

## 3 Results

### 3.1 Patient characteristics

204 patients with LA-ESCC who did not achieve pCR after NICT were enrolled in this study. The median follow-up time was 27.1 months in the PORT group and 27.0 months in the non-PORT group. Before matching, the baseline characteristics were unbalanced between the two groups. After PSM, there were expected balances of covariates in the two groups (**Table 1**).

**Table 1:** Baseline characteristics.

Variables	Before PSM			After PSM		
	Non-PORT (n = 154)	PORT (n = 50)	P-value	Non-PORT (n = 61)	PORT (n = 37)	P-value
Sex, n(%)			0.767			0.252
Female	21 (13.6)	6 (12.0)		14 (22.9)	5 (13.5)	
Male	133 (86.4)	44 (88.0)		47 (77.1)	32 (86.5)	
Age, n(%)			0.138			0.561
<60	50 (32.5)	22 (44.0)		18 (29.5)	13 (35.1)	
≥35	104 (67.5)	28 (56.0)		43 (70.5)	24 (64.9)	
BMI, n(%)			0.928			0.876
<20	36 (23.4)	12 (24.0)		14 (23.0)	9 (24.3)	
≥24	118 (76.6)	38 (76.0)		47 (77.0)	28 (75.7)	
KPS, n(%)			0.897			0.812
<90	97 (63.0)	32 (64.0)		41 (67.2)	24 (64.9)	
≥64	57 (37.0)	18 (36.0)		20 (32.8)	13 (35.1)	
Smoking, n(%)			0.424			0.965
No	55 (35.7)	21 (42.0)		25 (41.0)	15 (40.5)	
Yes	99 (64.3)	29 (58.0)		36 (59.0)	22 (59.5)	
Drinking, n(%)			0.839			0.211
No	61 (39.6)	19 (38.0)		31 (50.8)	14 (37.8)	
Yes	93 (60.4)	31 (62.0)		30 (49.2)	23 (62.2)	
Location, n(%)			0.432			0.615
Upper thoracic	7 (4.5)	3 (6.0)		2 (3.3)	2 (5.4)	
Middle thoracic	57 (37.0)	23 (46.0)		25 (41.0)	18 (48.6)	
Lower thoracic	90 (58.4)	24 (48.0)		34 (55.7)	17 (46.0)	
Differentiation, n(%)			0.666			0.665
Moderate-Well	97 (73.5)	33 (70.2)		42 (68.9)	27 (73.0)	
Poorly	35 (26.5)	14 (29.8)		19 (31.1)	10 (27.0)	
cStage, n(%)			0.849			0.976
II	41 (26.6)	14 (28.0)		15 (24.6)	9 (24.3)	
III-IV	113 (73.4)	36 (72.0)		46 (75.4)	28 (75.7)	
ypT, n(%)			<0.001			0.952
1-2	103 (66.9)	19 (38.0)		26 (42.6)	16 (43.2)	
3-4	51 (33.1)	31 (62.0)		35 (57.4)	21 (56.8)	
ypN, n(%)			0.044			0.707
0-1	128 (83.1)	35 (70.0)		45 (73.8)	26 (70.3)	
2-3	26 (16.9)	15 (30.0)		16 (26.2)	11 (29.7)	
ypN Status, n(%)			<0.001			0.640
ypN-	91 (59.1)	14 (28.0)		26 (42.6)	14 (37.8)	
ypN+	63 (40.9)	36 (72.0)		35 (57.4)	23 (62.2)	
ypStage, n(%)			<0.001			0.463
I-II	91 (59.1)	13 (26.0)		26 (42.6)	13 (35.1)	
III-IVA	63 (40.9)	37 (74.0)		35 (57.4)	24 (64.9)	
Down staging of T stage, n(%)			<0.001			0.965
No	51 (33.1)	32 (64.0)		36 (59.0)	22 (59.5)	
Yes	103 (66.9)	18 (36.0)		25 (41.0)	15 (40.5)	
Downstaging of N stage, n(%)			<0.001			0.937
No	53 (34.4)	33 (66.0)		34 (55.7)	22 (59.5)	
Yes	70 (45.5)	12 (24.0)		18 (29.5)	10 (27.0)	
Persistent N0	31 (20.1)	5 (10.0)		9 (14.8)	5 (13.5)	
Down staging of TNM stage, n(%)			<0.001			0.834
No	71 (46.1)	41 (82.0)		45 (73.8)	28 (75.7)	
Yes	83 (53.9)	9 (18.0)		16 (26.2)	9 (24.3)	
TRG, n(%)			0.007			0.951
0	22 (16.5)	4 (8.2)		2 (3.3)	2 (5.4)	
1	36 (27.1)	4 (8.2)		7 (11.5)	4 (10.8)	
2	48 (36.1)	24 (49.0)		32 (52.5)	20 (54.1)	
3	27 (20.3)	17 (34.6)		20 (32.7)	11 (29.7)	

PORT, Postoperative Radiotherapy; PSM, Propensity Score Matching; BMI, Body Mass Index; KPS, Karnofsky Performance Status; cStage, Clinical Stage; ypT, Pathological Tumor Stage after Neoadjuvant Therapy; ypN, Pathological Node Stage after Neoadjuvant Therapy; ypN+, Pathological staging N positive; ypStage, Pathological Stage after Neoadjuvant Therapy; TRG, Tumor Regression Grade.

### 3.2 Failure pattern analysis

**Figure 2:**
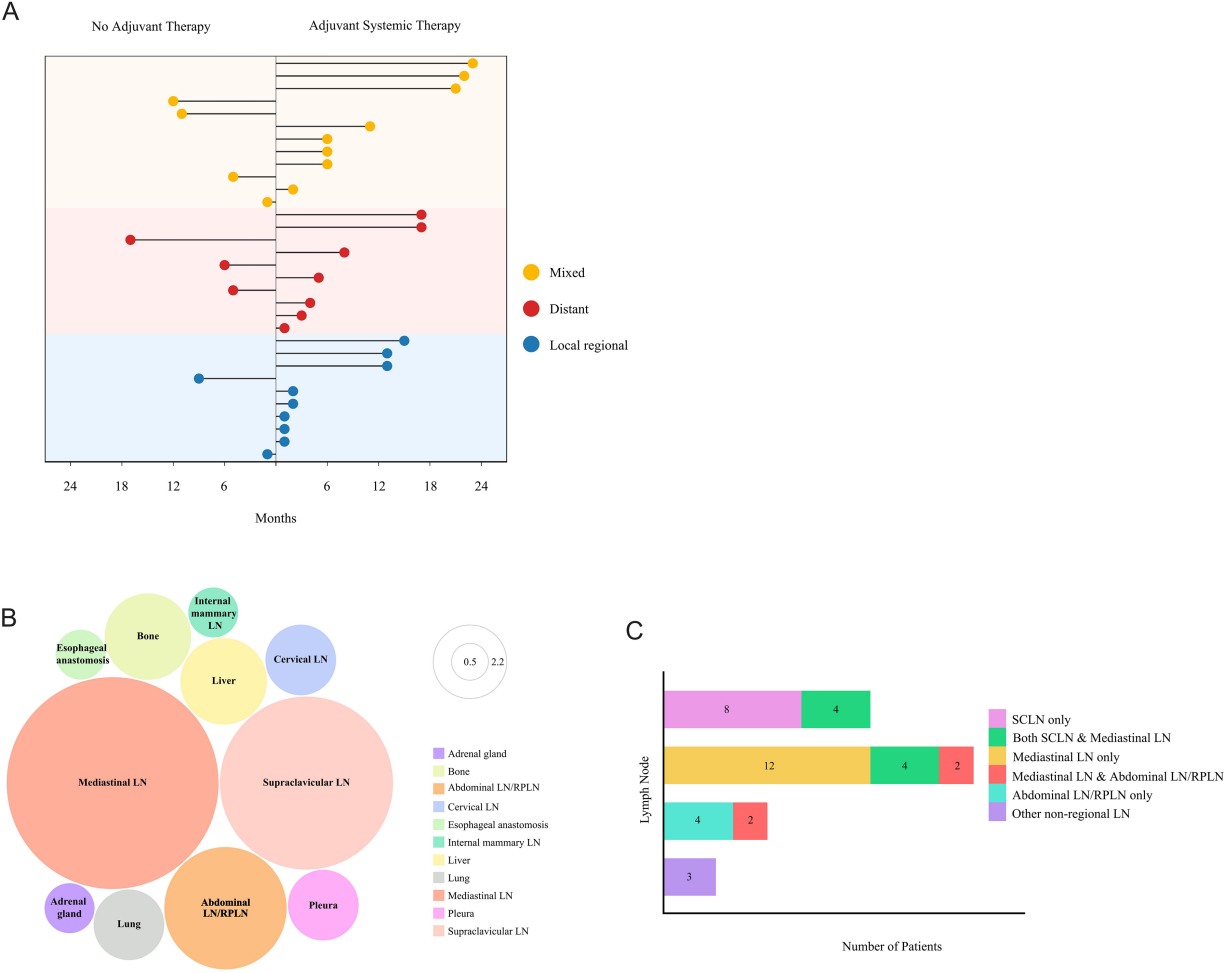
Recurrence patterns in LA-ESCC patients with non-pCR after NICT. **(A)** Dot plot displaying the recurrence patterns in patients without PORT including no adjuvant therapy and adjuvant systemic therapy. Yellow, mixed patterns including both locoregional recurrence and distant metastasis; red, distant metastasis only; blue, locoregional recurrence only. **(B)** Bubble chart showing the distribution of progression sites. LN, lymph node; Abdominal LN/RPLN, abdominal lymph node/retroperitoneal lymph node. **(C)** Lymph node metastasis patterns by Stacked bar chart. LN, lymph node; SCLN, supraclavicular lymph node; Abdominal LN/RPLN, abdominal lymph node/retroperitoneal lymph node.

Among the 154 non-pCR patients without PORT, recurrence developed in 32 (20.8%). As shown in the bubble chart (**Figure 2A**), locoregional recurrence (2/9, 22.2%), distant metastasis (3/9, 33.3%) and mixed pattern (4/9, 44.4%) were identified in non-adjuvant therapy patients. In the adjuvant systemic therapy group, the recurrence patterns included 34.8% (8/23) locoregional recurrence, 30.4% (7/23) distant metastasis, and 34.8% (8/23) mixed pattern. Additionally, 71.9% of these patients experienced recurrence within 1 year, 100% within 2 years, and the median time was 6.6 months. All recurrence sites were shown in **Figure 2B**. The locoregional recurrence pattern was predominantly characterized by regional lymph node recurrence, especially mediastinal lymph node metastasis, with only one case of anastomotic recurrence. Among distant metastases, supraclavicular lymph node metastasis was the most common pattern. Analysis of recurrent lymph nodes in **Figure 2C** revealed that mediastinal lymph node relapse was the primary failure pattern (46.1%), followed by supraclavicular lymph nodes (30.8%), abdominal/retroperitoneal lymph nodes (15.4%), and other non-regional lymph nodes (7.7%).

### 3.3 DFS analysis before and after PSM

**Figure 3:**
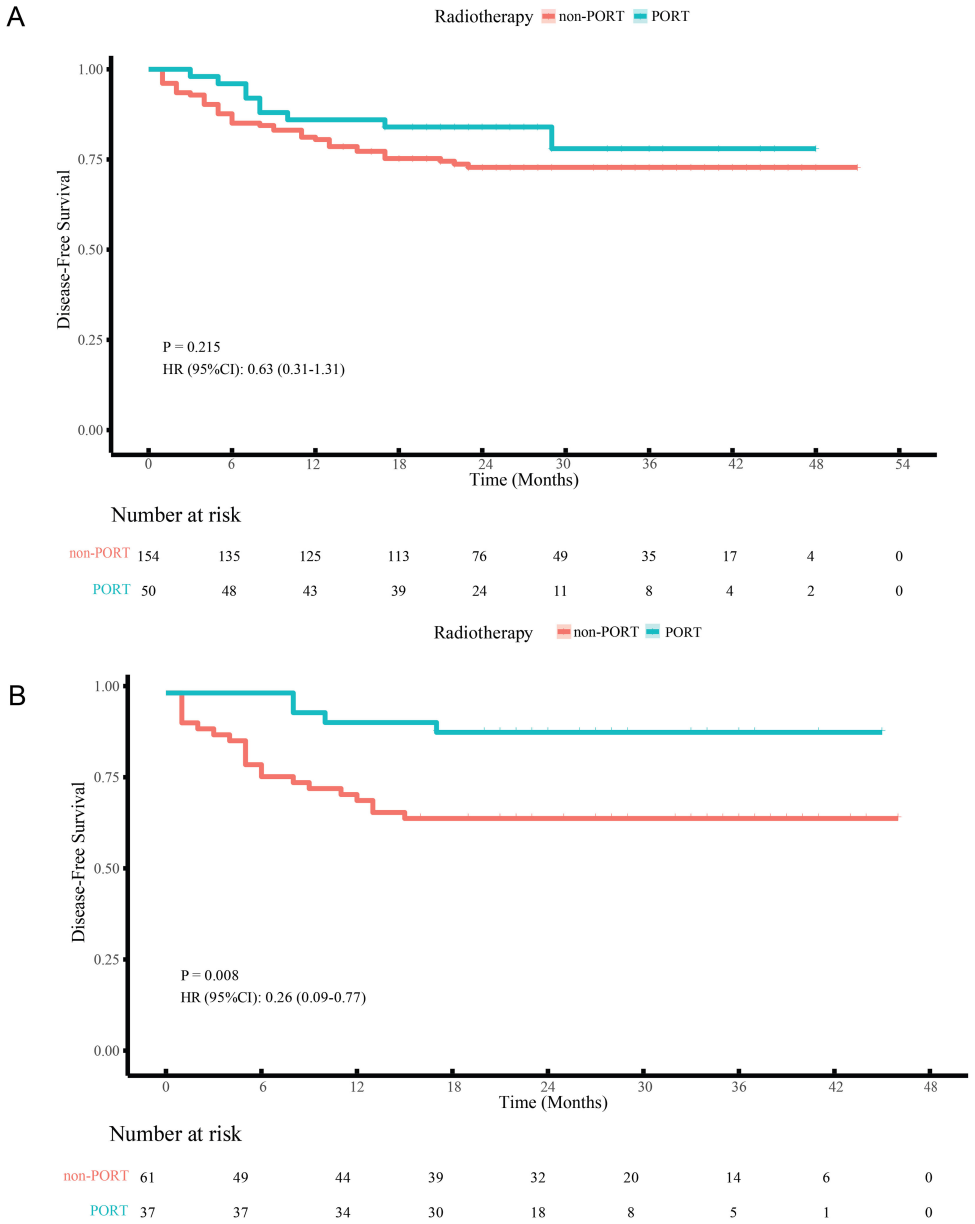
DFS curves of LA-ESCC patients with non-pCR after NICT before **(A)** and after **(B)** PSM. PORT, postoperative radiotherapy.

Before matching, KM analysis indicated no significant differences in DFS for PORT versus non-PORT (HR, 0.63; 95% CI, 0.31-1.31; P = 0.215) (**Figure 3A**). Univariable and multivariable Cox regression analyses in the non-PORT group identified ypN+ (HR, 2.54; 95% CI, 1.17-5.52; P = 0.018), TRG2 (HR, 3.95; 95% CI, 1.28-12.21; P = 0.017) and TRG3 (HR, 7.51; 95% CI, 2.38-23.66; P < 0.001) as independent risk factors for DFS (**Table 2**). In contrast, no such associations were observed in the PORT group (**Table 3**). Baseline characteristics showed significant imbalances in key prognostic factors before PSM, including ypT stage, ypN positivity, T/N/TNM downstaging, and tumor regression grade (TRG). After PSM, the two groups achieved balance in all prespecified covariates, and DFS was found to be significantly improved with PORT (HR, 0.26; 95% CI, 0.09-0.77; P = 0.008) (**Figure 3B**).

**Table 2:** Univariable and multivariable Cox for DFS in non-PORT patients.

Variables	Univariable Cox		Multivariable Cox	
	P-value	HR (95%CI)	P-value	HR (95%CI)
Sex				
Female		1.00 (Reference)	-	-
Male	0.413	0.71 (0.32-1.61)	-	-
Differentiation				
Moderate-Well		1.00 (Reference)	-	-
Poorly	0.086	1.79 (0.92-3.48)	-	-
Age (years)				
<60		1.00 (Reference)	-	-
≥Re	0.573	0.83 (0.44-1.57)	-	-
BMI (kg/m²)				
<20		1.00 (Reference)	-	-
≥Re	0.053	0.53 (0.28-1.01)	-	-
ypT				
1-2		1.00 (Reference)	-	-
3-4	<.001	3.44 (1.85-6.42)	-	-
ypN Status				
ypN-		1.00 (Reference)		1.00 (Reference)
ypN+	0.003	2.60 (1.39-4.88)	0.018	2.54 (1.17-5.52)
Downstaging of N stage				
No		1.00 (Reference)	-	-
Yes	0.116	0.59 (0.31-1.14)	-	-
Persistent N0	0.057	0.38 (0.14-1.03)	-	-
Downstaging of TNM stage				
No		1.00 (Reference)	-	-
Yes	0.248	0.70 (0.38-1.29)	-	-
TRG				
0-1		1.00 (Reference)		1.00 (Reference)
2	0.005	4.94 (1.62-15.00)	0.017	3.95 (1.28-12.21)
3	<0.001	11.01 (3.64-33.28)	<0.001	7.51 (2.38-23.66)

Univariable Cox, Univariable Cox Proportional Hazards Model; Multivariable Cox, Multivariable Cox Proportional Hazards Model; HR, Hazard Ratio; 95%CI, 95% Confidence Interval; BMI, Body Mass Index; ypT, Pathological Tumor Stage after Neoadjuvant Therapy; ypN+, Pathological staging N positive; TRG, Tumor Regression Grade.

**Table 3:** Univariable Cox for DFS in PORT patients.

Variables	Univariable Cox	
	P-value	HR (95%CI)
Differentiation		
Moderate-Well		1.00 (Reference)
Poorly	0.357	1.86 (0.50-6.94)
Age (years)		
<60		1.00 (Reference)
≥Re	0.185	0.39 (0.10-1.57)
BMI (kg/m²)		
<20		1.00 (Reference)
≥Re	0.388	2.50 (0.31-19.99)
ypT		
1-2		1.00 (Reference)
3-4	0.756	1.25 (0.31-5.00)
ypN		
0-1		1.00 (Reference)
2-3	0.085	3.18 (0.85-11.84)
Downstaging of N stage		
No		1.00 (Reference)
Yes	0.274	0.31 (0.04-2.51)
Persistent N0	0.998	NR (Not Reliable)
Downstaging of TNM stage		
No		1.00 (Reference)
Yes	0.999	NR (Not Reliable)
TRG		
0-1		1.00 (Reference)
2	0.237	0.31 (0.04-2.19)
3	0.814	1.22 (0.24-6.31)

Univariable Cox, Univariable Cox Proportional Hazards Model; HR, Hazard Ratio; CI, Confidence Interval; BMI, Body Mass Index; ypT, Pathological Tumor Stage after Neoadjuvant Therapy; ypN, Pathological Node Stage after Neoadjuvant Therapy; TRG, Tumor Regression Grade; NR, Not Reliable.

### 3.4 Subgroup analysis after PSM

**Figure 4:**
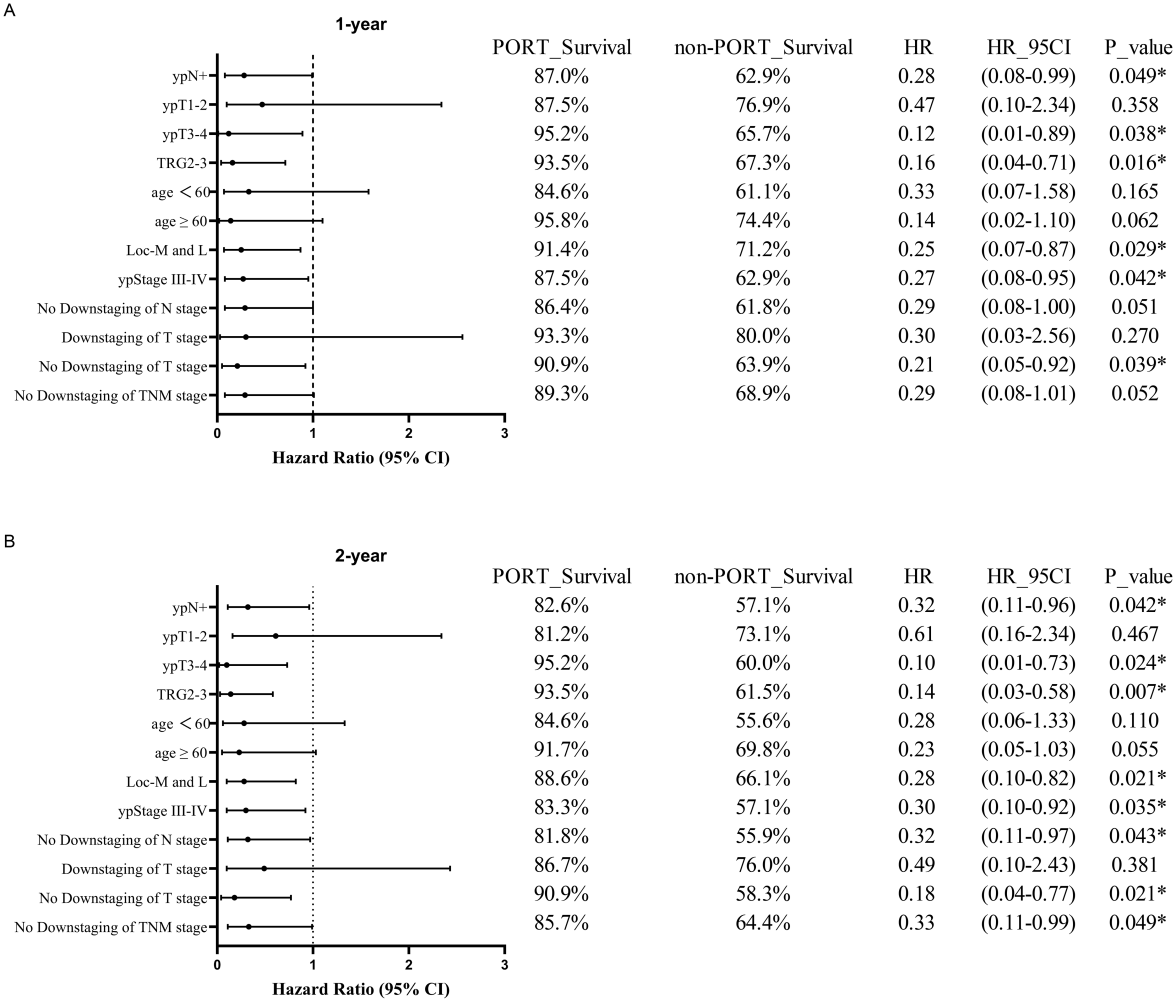
Forest plots of subgroup analysis in DFS between PORT and non-PORT groups after PSM at 1-year **(A)** and 2-year **(B)** follow-up. PORT, postoperative radiotherapy; HR, hazard ratio; CI, confidence interval; TRG, tumor regression grade; Loc-M and L, middle and lower thoracic tumor location; TNM, tumor-node-metastasis.

Subgroup analyses further identified specific populations benefiting from PORT. Significant 1-year and 2-year DFS rate improvements were observed in the ypN+ (1-year DFS, 87.0% vs. 62.9%; HR, 0.28; 95% CI, 0.08-0.99; P = 0.049; 2-year DFS, 82.6% vs. 57.1%; HR, 0.32; 95% CI, 0.11-0.96; P = 0.042), ypT3-4 (1-year DFS, 95.2% vs. 65.7%; HR, 0.12; 95% CI, 0.01-0.89; P = 0.038; 2-year DFS, 95.2% vs. 60.0%; HR, 0.10; 95% CI, 0.01-0.73; P = 0.024), TRG2-3 (1-year DFS, 93.5% vs. 67.3%; HR, 0.16; 95% CI, 0.04-0.71; P = 0.016; 2-year DFS, 93.5% vs. 61.5%; HR, 0.14; 95% CI, 0.03-0.58; P = 0.007), ypStage III-IVA (1-year DFS, 87.5% vs. 62.9%; HR, 0.27; 95% CI, 0.08-0.95; P = 0.042; 2-year DFS, 83.3% vs. 57.1%; HR, 0.30; 95% CI, 0.10-0.92; P = 0.035), non-downstaging of T stage (1-year DFS, 90.9% vs. 63.9%; HR, 0.21; 95% CI, 0.05-0.92; P = 0.039; 2-year DFS, 90.9% vs. 58.3%; HR, 0.18; 95% CI, 0.04-0.77; P = 0.021) and tumors located in the middle/lower thoracic esophagus (1-year DFS, 91.4% vs. 71.2%; HR, 0.25; 95% CI, 0.07-0.87; P = 0.029; 2-year DFS, 88.6% vs. 66.1%; HR, 0.28; 95% CI, 0.10-0.82; P = 0.021). Additionally, significant 2-year DFS improvements were observed in non-downstaging of N stage (2-year DFS, 81.8% vs. 55.9%; HR, 0.32; 95% CI, 0.11-0.97; P = 0.043) and non-downstaging of TNM stage (2-year DFS, 85.7% vs. 64.4%; HR, 0.33; 95% CI, 0.11-0.99; P = 0.049) (**Figure 4**).

### 3.5 Adverse events and safety profile

In a cohort of 204 patients receiving NICT, 23 were excluded due to missing data, resulting in 181 patients being included for adverse event analysis. A total of 158 patients (87.3%) experienced adverse events, the majority of which were grade I-II reactions. Among the entire cohort, grade 3–4 adverse events included myelosuppression in 18 cases (9.94%), grade 3 gastrointestinal reactions in 1 case, and immune-related organ injury in 2 cases. In the PORT subgroup, 8 (16.3%) patients developed esophageal mucosa injury, and 24 (49.0%) developed grade I-II myelosuppression. Notably, esophageal mucosa injury was significantly more common in the PORT group (P < 0.001). Grade I-II myelosuppression trended toward a higher incidence in the PORT group compared to the non-PORT group (P = 0.050), while grade I-II gastrointestinal reactions occurred more frequently in the non-PORT group (p=0.027) (**Table 4**), which might be due to the fact that some patients had received systemic therapy.

**Table 4:** Treatment-related complications: PORT vs non-PORT in LA-ESCC non-pCR post-NICT.

Complication	PORT (n)	PORT incidence (%)	Non-PORT (n)	Non-PORT incidence (%)	P-value
Myelosuppression I-II	24	49.0%	42	31.8%	0.050
Myelosuppression III-IV	6	12.2%	12	9.1%	0.578
Gastrointestinal reaction I-II	20	40.8%	80	60.6%	0.027
Gastrointestinal reaction III	0	0.0%	1	0.8%	1.000
Pneumonia	1	2.0%	2	1.5%	1.000
Hyponatremia	0	0.0%	2	1.5%	1.000
Immune-Related Pneumonitis	0	0.0%	1	0.8%	1.000
Immune-Related Hepatitis	0	0.0%	1	0.8%	1.000
Skin Reaction	1	2.0%	1	0.8%	0.469
Esophageal mucosa injury	8	16.3%	0	0.0%	< 0.001
Esophageal Anastomotic Fistula	1	2.0%	0	0.0%	0.271
No treatment-related complications	5	10.2%	18	13.6%	0.715

PORT, Postoperative Radiotherapy.

## 4 Discussion

In this study, our analysis revealed several central positive findings regarding the role of PORT in LA-ESCC patients without pCR following NICT. In patients not receiving PORT, the primary cause of treatment failure remains mediastinal and supraclavicular lymph node metastasis. After PSM to balance the baseline, PORT was found to be associated with a significant improvement in DFS, and subgroup analyses further identified the population that could potentially benefit, demonstrating that the DFS of PORT was predominantly prolonged in patients with high-risk clinicopathological features or poor response to NICT. With mainly low-grade adverse events, PORT showed acceptable feasibility.

A substantial body of research has demonstrated the significant efficacy of NICT in improving the prognosis of patients with resectable esophageal cancer. A systematic review and meta-analysis showed that this therapy achieved a pCR rate of 31.4% and a major pathological response (MPR) rate of 48.9% (15). A recent systematic review and network meta-analysis further corroborated the superior clinical efficacy and safety of NICT, highlighting the advantages of the camrelizumab plus TP regimen in terms of pCR, MPR, and R0 resection rates (16). Nevertheless, the CheckMate 577 trial established the principle that patients who do not achieve pCR after neoadjuvant therapy constitute a high-risk population requiring intensive adjuvant treatment (11). However, the study by Wu et al. indicated that even among the overall population receiving NICT combined with adjuvant immunotherapy, the 24-month DFS rate in non-pCR patients was significantly lower than that in pCR patients (77.3% vs. 100%) (17). Another study in the field of lung cancer has further confirmed that failure to achieve pCR after neoadjuvant immunotherapy is a key factor affecting prognosis, and among these patients, the cases with ypN2 have a mediastinal recurrence risk as high as 25% (18). However, there are few studies that have specifically focused on the LA-ESCC patients receiving NICT without pCR, and their failure patterns remain poorly characterized. In our study, we analyzed the failure patterns of this population and found that among all recurrence patterns, the mixed mode that involves both locoregional recurrence and distant metastasis was the predominant one. Among them, mediastinal lymph nodes and supraclavicular lymph nodes were the most frequent sites. These findings suggest that radiotherapy may serve as an effective strategy to reduce both locoregional and distant failure.

Although the NCCN guidelines do not recommend routine adjuvant therapy after R0 resection for ESCC (19), clinical investigation has reported a potential survival advantage with the use of PORT (20). PORT has been demonstrated to enhance local control by targeting the tumor bed and high-risk lymph nodes, thereby eliminating microscopic residual lesions and reducing the risk of local recurrence (21–23). Our study also found that in LA-ESCC patients who did not achieve pCR after NICT, ypN+ and TRG were identified as independent risk factors for DFS, whereas no significant prognostic association of these factors was observed in patients who received PORT. This suggests that radiotherapy may reduce the adverse impact of ypN+ and higher TRG on prognosis. To eliminate the impact of baseline differences, we performed PSM analysis, which further indicated that PORT significantly improved DFS in this patient population.

Given the demonstrated benefit of PORT, further subgroup analyses aimed to clarify which specific patients derive the greatest benefit. Postoperative lymph node staging after NICT has been identified as a significant predictor of patient prognosis (24). In our study, for patients with poor pathological stages, such as ypN+, ypT3-4, ypStage III-IVA, the addition of PORT can result in DFS benefit. This might be due to the fact that this population has a high risk of mediastinal and supraclavicular lymph node metastasis, and the application of radiotherapy contributes to the increase of the local control rate, thereby improving the prognosis. Additionally, we found that for patients with poor response to NICT (e.g., TRG 2-3, no ypT/ypN/ypTNM downstaging), PORT also significantly improved DFS. This poor outcome after NICT may be attributed to high tumor burden and tumor immunosuppressive microenvironment (TIME) before treatment. However, the addition of PORT can directly eliminate tumor cells or invisible microscopic residual lesions via high-energy rays, thereby reducing the risk of subsequent recurrence and metastasis. Furthermore, radiotherapy can also remodel the TIME and elicit systemic anti-tumor immunity (25), which ultimately translates into the improvement of DFS benefits.

A comprehensive analysis of 27 NICT trials revealed that the incidence of treatment-related serious adverse events (trSAEs) was 26.9%, with no treatment-related deaths reported, suggesting that this treatment is safe and well-tolerated (15). Additionally, a study by Zhang et al. on PORT for ESCC showed that acute toxicities were predominantly mild, with grade 3 toxicities mainly consisting of leukopenia and no severe skin, cardiac, or pulmonary toxicities or treatment-related deaths observed (21). In our study, patients who received PORT after NICT mainly experienced mild toxicities, further confirming the favorable tolerability of this combined treatment regimen. This also reminds us that in clinical practice, emphasis should be placed on the prevention and management of radiation esophagitis and myelosuppression.

This study has several limitations. First, the relatively small sample size may have reduced the statistical power and limited the inclusion of potential confounders in the multivariate analysis. Second, the relatively short follow-up period prevents a comprehensive assessment of long-term outcomes, as the duration of follow-up for some patients may not fully reflect their long-term prognosis. Finally, as a retrospective observational study, residual confounding such as from heterogeneous NICT regimens cannot be fully excluded despite PSM adjustment, and patient quality of life was unassessed. These limitations highlight the need for prospective studies with standardized PORT protocols to validate findings.

## 5 Conclusion

In LA-ESCC patients who do not achieve pCR after NICT, mediastinal and supraclavicular lymph node recurrence is the primary causes of treatment failure among those not receiving PORT. Significant DFS benefit is observed in patients with PORT, particularly in those with high-risk clinicopathological features or suboptimal response to NICT, and it exhibits a favorable safety profile. Our study confirmed the potential value of PORT in improving the short-term outcomes of patients, but future prospective large-sample clinical trials are still needed to further verify its significance.

## Data Availability

The original contributions presented in the study are included in the article/supplementary material. Further inquiries can be directed to the corresponding authors.
